# Ruthenacarborane and Quinoline: A Promising Combination for the Treatment of Brain Tumors

**DOI:** 10.3390/molecules26133801

**Published:** 2021-06-22

**Authors:** Dijana Drača, Milan Marković, Marta Gozzi, Sanja Mijatović, Danijela Maksimović-Ivanić, Evamarie Hey-Hawkins

**Affiliations:** 1 Department of Immunology, Institute for Biological Research “Siniša Stanković”, National Institute of Republic of Serbia, University of Belgrade, Bul. Despota Stefana 142, 11060 Belgrade, Serbia; dracadiana@gmail.com (D.D.); milan.markovic@ibiss.bg.ac.rs (M.M.); 2 Faculty of Chemistry and Mineralogy, Institute of Inorganic Chemistry, Leipzig University, Johannisallee 29, 04103 Leipzig, Germany; marta.gozzi@uni-leipzig.de; 3 Medical Faculty, Institute for Medical Physics and Biophysics, Leipzig University, Härtelstr. 16–18, 04107 Leipzig, Germany

**Keywords:** ruthenacarborane, autophagy, glucose deprivation, glioma

## Abstract

Gliomas and glioblastomas are very aggressive forms of brain tumors, prone to the development of a multitude of resistance mechanisms to therapeutic treatments, including cytoprotective autophagy. In this work, we investigated the role and mechanism of action of the combination of a ruthenacarborane derivative with 8-hydroxyquinoline (**8-HQ**), linked via an ester bond (complex **2**), in rat astrocytoma C6 and human glioma U251 cells, in comparison with the two compounds alone, i.e., the free carboxylic acid (complex **1**) and **8-HQ**, and their non-covalent combination ([**1** + **8-HQ**], in 1:1 molar ratio). We found that only complex **2** was able to significantly affect cellular viability in glioma U251 cells (IC_50_ 11.4 μM) via inhibition of the autophagic machinery, most likely acting at the early stages of the autophagic cascade. Contrary to **8-HQ** alone, complex **2** was also able to impair cellular viability under conditions of glucose deprivation. We thus suggest different mechanisms of action of ruthenacarborane complex **2** than purely organic quinoline-based drugs, making complex **2** a very attractive candidate for evading the known resistances of brain tumors to chloroquine-based therapies.

## 1. Introduction

From a chemical biology perspective, little is known about the mechanisms of action of seemingly biologically active (metalla)carborane-containing compounds, particularly in terms of effects on physiological and pathophysiological biochemical processes at the cellular and sub-cellular level [1,2,3]. Chemical conjugation of icosahedral *closo*-carboranes, metallacarboranes and *nido*-carborates(−1) to an organic or macromolecular substrate is a widely explored approach in medicinal chemistry [4,5,6,7], which has led to the development of entire libraries of compounds that often show either superior biological activities or substantially different activities than their respective purely organic or organometallic counterparts [8,9,10,11]. Nonetheless, what triggers the activity of a cluster-containing potential drug at the biomolecular level in a specific biological environment and how, remains largely unknown and is hardly investigated.

**Figure 1:**
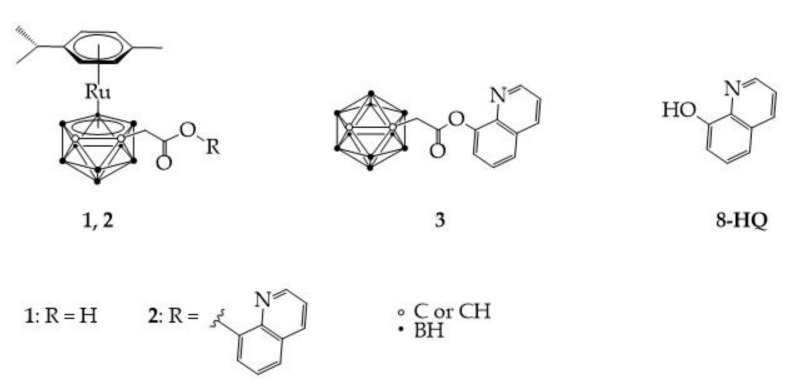
Compounds investigated in the present study. Compounds **1**–**3** were previously reported by us [13], **8-HQ** is commercially available.

We have recently published an extensive review on (metalla)carboranes as “new” nanomedicine platforms [12], urging the boron community to consistently treat such compounds as potential colloidal aggregators in aqueous environment, and as such to implement standardized and homogeneous biophysical and biological tests. In this short communication, we focus our attention on further biological testing of a small series of compounds (see Figure 1), using a consistent and complementary approach to our earlier investigations [13]. In fact, we had shown that ruthenacarborane complex **2**, which contains a quinolyl residue, displays a dual mode of action in vitro against human glioblastoma LN229 cells, namely it strongly inhibited proliferation and cytoprotective autophagy, with IC_50_ values in the same concentration range as for the clinically approved drug chloroquine, which is unfortunately often associated with the development of drug resistance mechanisms [14,15,16]. Here, we aim at investigating further the role and mechanism of action of the combination of a ruthenacarborane derivative with hydroxyquinoline, i.e., the aromatic building block of chloroquine, in two different types of brain tumor cells, namely rat astrocytoma (C6) and human glioma (U251) cells, using analogous testing protocols to those used with the LN229 cell line.

We considered it to be important to test our promising compound against other types of brain tumor cells, because there is extensive evidence in the literature that gliomas and glioblastomas are morphologically and functionally very heterogeneous tumors [17,18], often displaying a complex network of intercellular cross-talks, very difficult to tackle therapeutically, due to the insurgence of several parallel mechanisms of resistance.

To this end, we designed the experiments with different combinations of the compounds shown in Figure 1, namely **1**, **2**, **3** and **8-HQ** alone, and a 1:1 molar ratio of **1** and **8-HQ**, in order to investigate the effects of each potentially biologically active species, as well as possible additive or synergistic effects. Each compound or combinations thereof were tested in in vitro cell viability assays against the two cell lines C6 and U251, as well as via fluorescence-based assays, to study the effect on cellular proliferation and acidic vesicle formation, as an indication of the occurrence of autophagic events. Finally, the effects of glucose deprivation on the activity of complex **2** and **8-HQ** against the U251 cell line were also investigated. It is important to point out that the investigated compounds (specifically **2**, **3** and the combination of **1** with **8-HQ**) were not designed to target a specific binding pocket of a receptor or enzyme, as is often done in drug design strategies, but rather to possibly tackle and affect the complex cellular autophagic machinery with therapeutic purposes. The focus of this work is on brain tumor cell lines due to consistency with our previous studies [13] and also because there is convincing evidence that boron cluster-containing compounds have great potential as therapeutics to target the central nervous system [6,19,20,21,22], since their highly hydrophobic nature is expected to strongly promote blood-brain-barrier penetration.

## 2. Results

Previously, we reported that complex **2** strongly inhibited cellular division and motility in human glioblastoma LN229 cells in vitro [13]. Moreover, **2** also inhibited expression of microtubule-associated protein light chain 3B (LC3B), which is one of the established biomarkers of the cellular autophagic machinery, since it forms a stable association complex with the membrane of autophagosomes. This dual mode of action of **2** is very promising for the treatment of autophagy-prone cancers, where autophagic processes often contribute to the establishment and/or development of drug resistances, such as in brain tumors of the glioma and glioblastoma types [23]. 

The motivation of using C6 and U251 cell lines for the present study is as follows. The C6 rat astrocytoma cell line is a well-established model for brain tumors [24] because it simulates the high growth rate, vascularization and infiltrative character of glioblastoma multiforme well; however, conclusions drawn from studies performed on non-human biological material should always be treated with diligence due to obvious inter-species differences. The U251 human glioma cell line is also a broadly investigated model, occurs often in brain tumor tissues, but bears quite a few differences with respect to the LN229 cell line [18], e.g., different morphology, proliferation rate, deformability and motility; it can thus provide a broader picture of the therapeutic effect of a ruthenacarborane-based treatment, in the context of high tumor heterogeneity.

From a strictly chemical point of view, complex **2** contains an ester bond between the ruthenacarborane moiety and the quinolyl residue, which allows the release of the quinolyl molecule over 2.5 h under simulated biological conditions (37 °C, phosphate-buffered saline solution), as previously reported by us [13]. *closo*-Carborane derivative **3** is the structural and isoelectronic analogue of **2**, but does not contain the [Ru{*η*^6^-(*p*-cymene)}]^2+^ fragment. The combination of complex **1** with **8-HQ** is chemically analogous to complex **2**, albeit the two fragments are not covalently bound, which eliminates the effect of slow release of the quinolyl moiety and might thus produce different biological effects in the studied cells. The 8-hydroxyquinoline derivatives are widely recognized active molecules with a plethora of effects [25,26,27], including up- or down-regulation of cellular autophagic flux, according to the specific type of tumor, resulting in impaired cellular viability. Within this study, **8-HQ** represents the purely organic part of the hybrid complex **2**. Thus, treating the chosen cell lines with either of these compounds, and combinations thereof, in parallel can reveal important information on the role of each potentially active fragment.

**Figure 2:**
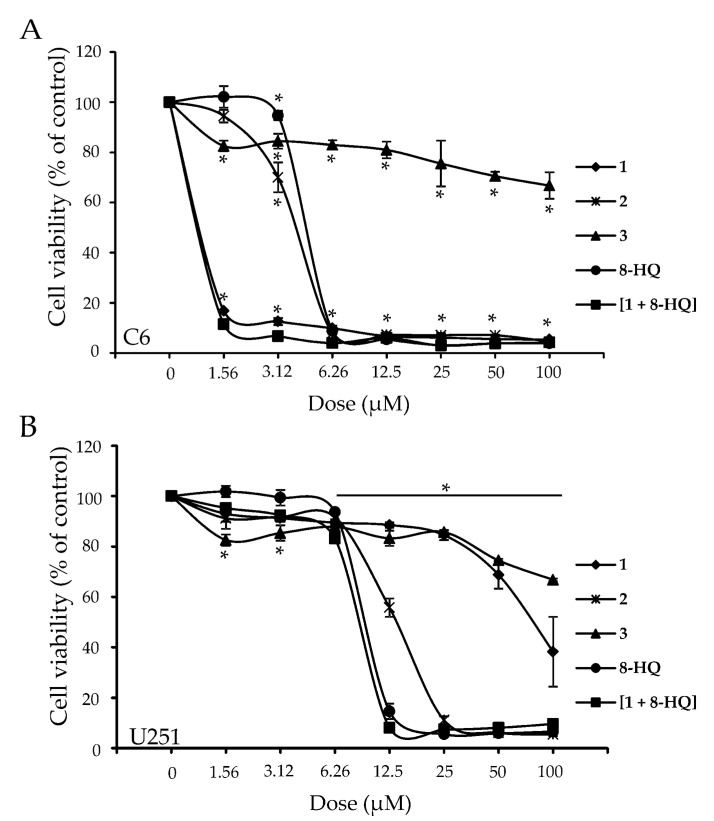
Cell viability curves of C6 (A) and U251 (B) cell lines in response to treatment with compounds **1**–**3**, **8-HQ** and [**1** + **8-HQ**]. Curves from CV staining protocol are presented. Experiments were run in three independent replicates. * indicates statistically significant (*p* < 0.05) values with respect to control (untreated cells). (CV: crystal violet).

Treatment of the C6 and U251 cell lines with compound **3** for 72 h showed no significant impairment of cell viability, for the tested concentration range (IC_50_ values higher than 100 μM; Table 1, Figure 2), while complex **2** was active against both cell lines in the low micromolar range. This strongly suggests that the organometallic fragment [Ru{*η*^6^-(*p*-cymene)}]^2+^ is needed for activity. While all other compounds were highly active against the C6 cell line, U251 cells showed remarkably higher resistance to treatment with **1** alone, i.e., the ruthenacarborane complex containing a free carboxylic acid group, in comparison to complex **2**, i.e., its quinolyl ester conjugate, as we had previously observed in the same cell line and in glioblastoma LN229 cells [13]. Both cell lines investigated here expressed similar sensitivity to **8-HQ** alone or in combined treatment ([**1** + **8-HQ**], Table 1, Figure 2).

**Figure 3:**
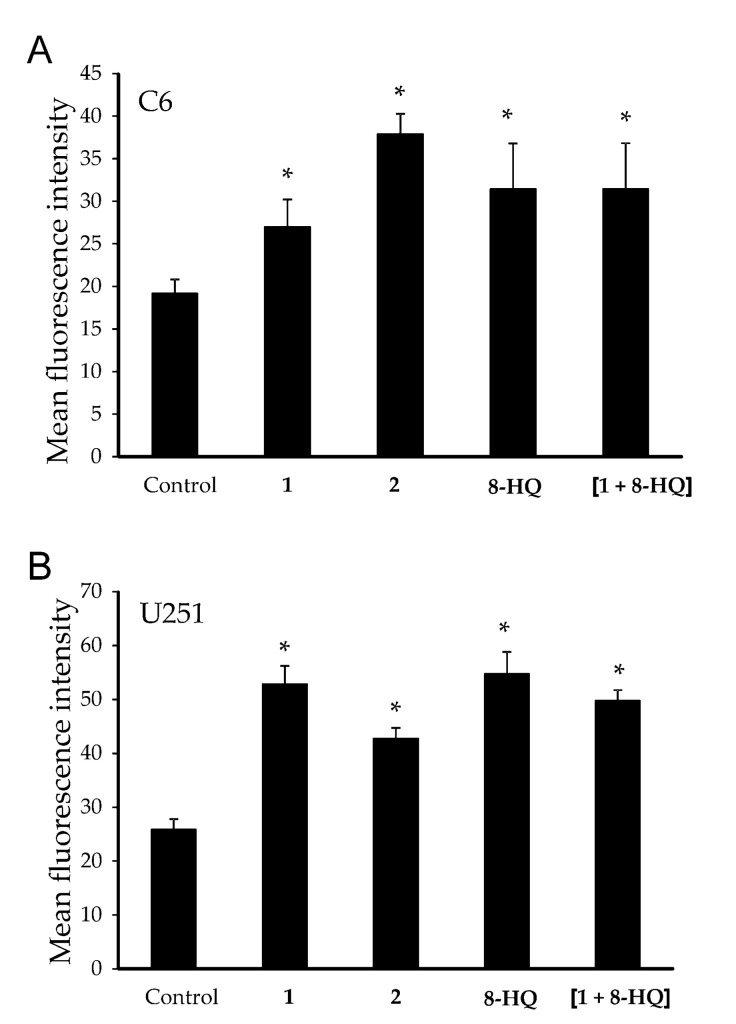
Green fluorescence emission values after CFSE staining of C6 (A) and U251 (B) cells, treated with IC_50_ doses of **1**, **2**, **8-HQ** and [**1** + **8-HQ**]. Mean fluorescence values from three independent replicates are shown. Data are presented as bar diagrams. * indicates statistically significant (*p* < 0.05) values with respect to control (untreated cells). (CFSE: carboxyfluorescein diacetate succinimidyl ester).

Flow cytometric analysis showed that all compounds significantly inhibited cell division (Figure 3), with respect to the control, when applied at the respective IC_50_ concentrations. This is similar to what we previously observed for complex **2** in LN229 cells [13], suggesting, at least for this molecule, a similar effect on cellular division also on other types of brain tumor cells.

**Figure 4:**
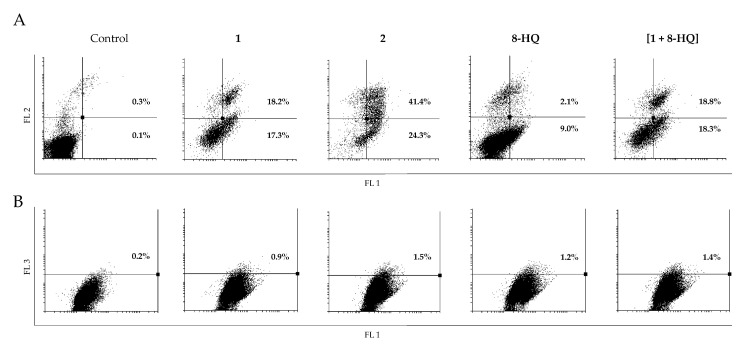
Flow cytometric analysis of C6 cells after AnnV/PI (panel A) and AO (panel B) staining, treated with IC_50_ doses of **1**, **2**, **8-HQ** and [**1** + **8-HQ**]. Representative dot-plots from three independent replicates are presented. (AnnV/PI: Annexin V/propidium iodide; AO: acridine orange; FL1: green channel; FL2: orange channel; FL3: dark red channel).

**Figure 5:**
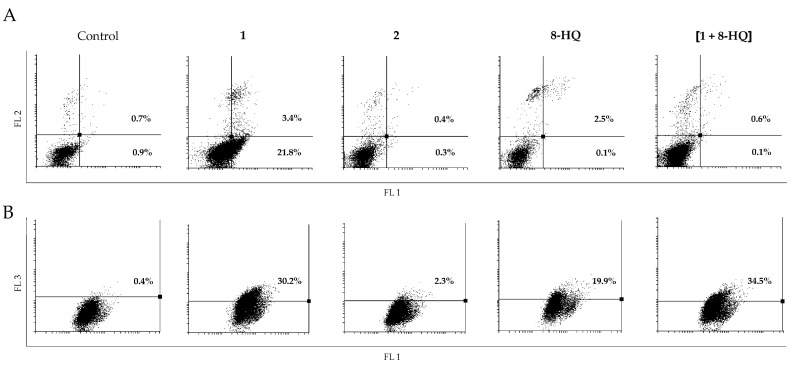
Flow cytometric analysis of U251 cells after AnnV/PI (panel A) and AO (panel B) staining, treated with IC_50_ doses of **1**, **2**, **8-HQ** and [**1** + **8-HQ**]. Representative dot-plots from three independent replicates are presented. (AnnV/PI: Annexin V/propidium iodide; AO: acridine orange; FL1: green channel; FL2: orange channel; FL3: dark red channel).

While in C6 cells inhibition of proliferation was accompanied by induction of apoptosis (Figure 4, panel A), in U251 cells only compound **1** enhanced the amount of early apoptotic cells (Figure 5, panel A). Acridine orange staining of C6 cells revealed only a slightly higher number of cells containing acidic vesicles upon treatment with **1**, **2**, **8-HQ** and [**1** + **8-HQ**] than in the control, but without showing a pattern within the series of investigated compounds (Figure 4, panel B). On the other hand, treatment of U251 cells with **1** promoted, in parallel to intensive apoptotic processes (Figure 5, panel A), a strong increase in the percentage of cells rich in acidic vesicles (Figure 5, panel B). An analogous effect was found upon treatment with **8-HQ** alone and [**1** + **8-HQ**] (Figure 5, panel B).

Conversely, exposure of U251 cells to complex **2** completely abolished this effect (Figure 5, panel B), confirming its ability to prevent formation of cytoplasmic acidic vesicles, either lysosomes or autolysosomes. Striking is that the mixture [**1** + **8-HQ**] does not have the same effects on suppression of formation of acidic vesicles as complex **2**, which suggests that the presence of an ester bond and/or the slow release of the quinolyl fragment from the ruthenacarborane complex are required for this effect.

Moreover, these results suggest that complex **2** affects the autophagic machinery in a different manner than the well-known autophagy inhibitor chloroquine and its derivatives, including 8-hydroxyquinoline. Namely, despite disagreements in the literature regarding the mechanisms of action of chloroquine and derivatives depending on the type of cells and tissue, generally chloroquine compromises lysosomal function and/or fusion with autophagosomes at the final stages of the autophagic machinery, leading to aberrant degradation processes [28,29,30]. Thus, this does not reflect on the number of acidic vesicles detected by acridine orange staining, and even amplifies it through lysosomal swelling. Complex **2** obviously inhibited autophagic processes through abrogated formation of acidic vesicles, suggesting that the mechanism of inhibition is switched to early steps in this process. This is somewhat in concordance with our previously published data about diminished expression of microtubule-associated protein light chain 3B (LC3B) upon treatment of LN229 cells with **2** [13], a protein which is involved in the early stage formation of autophagosomes [31]. Whether analogous mechanisms are at play also in U251 cells requires further testing, which is currently undergoing in our laboratories.

**Figure 6:**
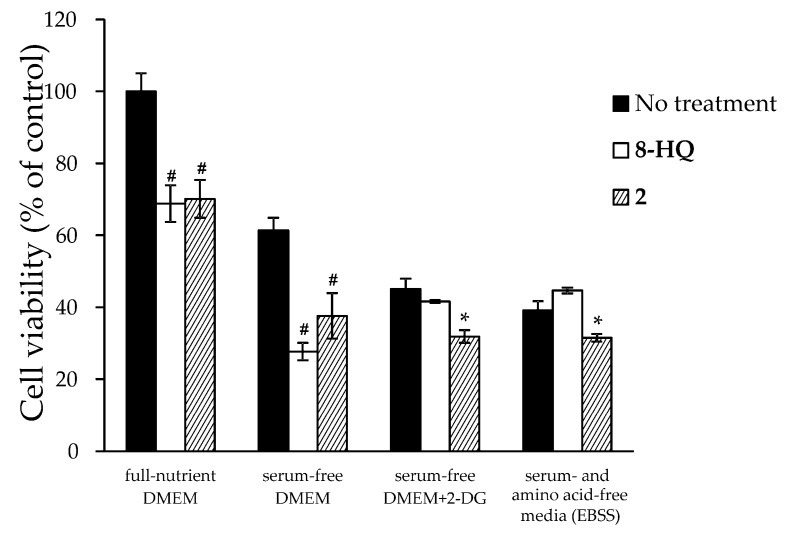
Cell viability (% of control) of U251 cells treated with **2** and **8-HQ**, applied at their respective IC_50_ values from Table 1 (CV assay). Cells were cultivated in full nutrient DMEM, serum-free DMEM with and without 2-DG, and essential EBSS medium without serum, glucose and amino acids. Cell viability was estimated by CV test after 24 h of incubation. Mean values with respective standard deviations from two independent replicates are presented. # indicates statistically significant (*p* < 0.05) values with respect to relevant control, while * indicates statistically significant in comparison to the treatment with **8-HQ** in serum-free DMEM without 2-DG. (DMEM: Dulbecco’s Modified Eagle´s Medium; EBSS: Earle´s Balanced Salt Solution; 2-DG: 2-deoxy-d-glucose; CV: crystal violet).

Lastly, the response of U251 cells upon treatment with **8-HQ** and ruthenacarborane complex **2** under glucose depletion conditions were investigated. Recently, strong evidence has in fact been collected that glucose metabolism plays a central role in the development of resistances to chloroquine in brain tumors: glucose depletion was, for example, found to completely abolish chloroquine-mediated cytotoxicity in UVW glioma cells [15]. Thus, U251 cells were cultivated under different conditions, namely i) full nutrient DMEM medium, ii) serum-free DMEM medium, with and without 2-deoxy-d-glucose (2-DG), a known glycolysis inhibitor, and iii) serum-, glucose- and amino acids-free essential EBSS medium. After 24 h incubation in serum-free DMEM and without 2-DG, cell viability dropped to about 30% and 40% upon treatment with **8-HQ** and **2**, respectively, compared to the control (Figure 6). Conversely, glycolysis blockade with 2-DG restored cell viability after treatment with **8-HQ**, with respect to the control, indicating that glucose depletion led to neutralization of the effect of **8-HQ**. Analogous results were observed in EBSS medium, in absence of glucose, serum and amino acids. On the other hand, glucose deprivation did not significantly impair the activity of complex **2** against U251 cells, either in serum-free DMEM or in essential EBSS (Figure 6). Thus, these experiments integrate our conclusions from fluorescence-based assays (see above), where it was observed that complex **2** seems to act through different mechanisms than purely organic quinoline-containing compounds against brain tumor cells in vitro, even though both molecules affect the autophagic machinery.

Surely, these preliminary studies are highly promising, since the different mechanisms of action of complex **2** against several types of human brain tumor cells might be of advantage for the treatment of gliomas and glioblastomas that show proclivity to evade (hydroxy)chloroquine-based therapies [15].

## 3. Materials and Methods

### 3.1. Chemicals

8-Hydroxyquinoline (**8-HQ**) was purchased from Merck KGaA (Darmstadt, Germany) and recrystallized from diethyl ether before use. Compounds **1**–**3** were synthesized and purified as previously reported [13].

### 3.2. Reagents and Cells

Fetal bovine serum (FBS), cell culture medium RPMI-1640, phosphate-buffered saline (PBS), dimethyl sulfoxide (DMSO), 3-(4,5-dimethylthiazol-2-yl)-2,5-diphenyl-tetra- zolium bromide (MTT), carboxyfluorescein diacetate succinimidyl ester (CFSE), crystal violet (CV), 2-deoxy-d-glucose (2-DG) and propidium iodide (PI) were purchased from Sigma Aldrich (St. Louis, MO, USA). Dulbecco’s Modified Eagle´s Medium (DMEM) and Earle´s Balanced Salt Solution (EBSS) were from Biological Industries (Beit HaEmek, Israel). The penicillin/streptomycin antibiotics solution was from Biological Industries (Cromwell, CT, USA). Acridine orange (AO) was obtained from Labo-Moderna (Paris, France). Annexin V-FITC (AnnV) was purchased from Santa Cruz Biotechnology (Dallas, TX, USA). Rat astrocytoma C6 and human glioma U251 cells were a kind gift from Dr. L. Harhaji-Trajković, Institute for Biological Research “Siniša Stanković”-National Institute of Republic of Serbia (Belgrade, Serbia). Cells were cultivated in HEPES-buffered (HEPES = 4-(2-hydroxyethyl)-1-piperazineethanesulfonic acid) RPMI-1640 medium supplemented with 10% FBS, 2 mM l-glutamine, 0.01% sodium pyruvate and antibiotics at 37 °C in a humidified atmosphere with 5% CO_2_. For viability assays, C6 and U251 cells were seeded at 5 × 10^3^ and 3 × 10^3^ density/well in 96-well plates, and for flow cytometry at 2 × 10^5^ and 1 × 10^5^ density/well in 6-well plates, respectively. 

### 3.3. Preparation of the Drug Solutions

DMSO stock solutions of **1**–**3** and **8-HQ** were prepared at concentrations of 50 mM, 57 mM, 30 mM and 40 mM, respectively, and kept at −20 °C. Working concentrations were made directly in cell culture medium. DMSO content was 0.2 vol%, in the highest tested concentration.

### 3.4. Cell Viability Assays

C6 and U251 cells were treated with various concentrations of **1**–**3**, **8-HQ** and a 1:1 molar ratio of **1** and **8-HQ** ([**1** + **8-HQ**]), up to 100 µM. Cells were incubated for 72 h. MTT and CV assays were performed as described elsewhere [13]. Experiments were run in three independent replicates. Cell viability is expressed relative to the control (untreated cells). 

For evaluation of the activity of complex **2** and **8-HQ** under conditions of glucose deprivation, two settings were employed. First, cells were treated with an IC_50_ concentration of **2** or **8-HQ** in serum-free DMEM medium, in the presence or absence of the glycolysis inhibitor 2-DG (final concentration 5 mM). Alternatively, cells were incubated in essential medium (EBSS) without glucose, serum and amino acids. Cell viability was estimated after 24 h by CV test. Experiments were run in two independent replicates.

### 3.5. CFSE Staining

To estimate the influence of the compounds on the cellular proliferation, cells were pre-stained with 1 μM CFSE solution for 10 min at 37 °C and then exposed to IC_50_ doses of **1**, **2**, **8-HQ** and [**1** + **8-HQ**] for 72 h. Finally, cells were detached, dissolved in PBS and analyzed with CyFlow^®^ Space Partec using the PartecFloMax^®^ software. Experiments were run in three independent replicates.

### 3.6. Annexin V-FITC/PI and AO Staining

Cells were exposed to the IC_50_ doses of **1**, **2**, **8-HQ** and [**1** + **8-HQ**] for 72 h. For detection of apoptosis, cells were harvested, washed with PBS and double stained with AnnV and PI (15 μg mL^−1^) according to the manufacturer’s protocol for 15 min at room temperature. For detection of autophagosomes, cells were stained with a 10 μM solution of AO for 15 min at 37 °C. Cells were then washed and resuspended in PBS, and finally analyzed using the PartecFloMax^®^ software. Experiments were run in three independent replicates.

### 3.7. Statistical Analysis 

Analysis of variance (ANOVA), followed by a Student-Newman–Keuls test, was used for significance of the differences between treatments, and a *p* value less than 0.05 was taken as statistically significant.

## 4. Conclusions

Inhibition of cytoprotective autophagy using chloroquine and its derivatives is considered one of the most promising approaches for chemosensitization of brain tumors of the glioma and glioblastoma types. However, due to its functional pleiotropy, based on a multitude of molecular targets [23], chloroquine efficiency can be highly affected and compromised in the context of advanced tumor microenvironment [32], which often provides conditions of hypoxia and lack of nutrition, including glucose. Here, we showed that complex **2**, which combines a ruthenacarborane scaffold with an 8-hydroxyquinoline moiety via an ester bond, impairs the cell viability of human U251 glioma cells, with analogous potency to **8-HQ** alone, i.e., the organic moiety of complex **2**, which consists of the aromatic building block (quinolyl group) of chloroquine. Moreover, complex **2** was able to effectively inhibit cytoprotective autophagy, likely via a different mechanism than that of quinolyl-containing organic scaffolds, i.e., inhibition of early stages of autophagosome formation, instead of late stages. Furthermore, the ability of complex **2** to impair cell viability also under glucose deprivation conditions, where **8-HQ** loses most of its activity, makes this compound a rather promising hybrid molecule for further investigations as potential therapeutic treatment for aggressive autophagy-prone brain tumors.

## Figures and Tables

**Table 1:** IC_50_ values for **1**–**3**, **8-HQ** and [**1** + **8-HQ**] from MTT and CV cell viability assays. Standard deviations for each IC_50_ value are given. Experiments were run in three independent replicates.

		IC_50_ (μM)	
Compound	Assay ^1^	C6	U251
**1**	MTT	0.8 ± 0.1	61.9 ± 11.4
	CV	1.0 ± 0.1	75.3 ± 7.6
**2**	MTT	3.3 ± 0.3	9.2 ± 1.0
	CV	14.4 ± 0.1	11.4 ± 3.9
**3**	MTT	>100	>100
	CV	>100	>100
**8-HQ**	MTT	3.8 ± 0.4	9.0 ± 0.8
	CV	4.7 ± 0.1	9.6 ± 0.2
[**1** + **8-HQ**] ^2^	MTT	0.9 ± 0.1	7.4 ± 0.2
	CV	1.0 ± 0.1	8.8 ± 0.2

^1^ MTT = 3-(4,5-dimethylthiazol-2-yl)-2,5-diphenyltetrazolium bromide; CV = crystal violet. ^2^ [**1** + **8-HQ**] indicates incubation of each cell line with a mixture of complex **1** and **8-HQ**, in 1:1 molar ratio.

## Data Availability

The data presented in this study are available on request from the corresponding author.

## References

[1] Murphy N., McCarthy E., Dwyer R., Farràs P. (2021). Boron clusters as breast cancer therapeutics. J. Inorg. Biochem..

[2] Stockmann P., Gozzi M., Kuhnert R., Sárosi M.-B., Hey-Hawkins E. (2019). New keys for old locks: Carborane-containing drugs as platforms for mechanism-based therapies. Chem. Soc. Rev..

[3] Zargham E.O., Mason C.A., Lee M.W. (2019). The use of carboranes in cancer drug development. Int. J. Cancer Clin. Res..

[4] Hey-Hawkins E., Viñas-Teixidor C. (2018). Boron-Based Compounds: Potential and Emerging Applications in Medicine.

[5] Leśnikowski Z.J. (2016). Challenges and opportunities for the application of boron clusters in drug design. J. Med. Chem..

[6] Barth R.F., Mi P., Yang W. (2018). Boron delivery agents for neutron capture therapy of cancer. Cancer Commun..

[7] Grimes R.N. (2016). Carboranes.

[8] Kuhnert R., Sárosi M.-B., George S., Lönnecke P., Hofmann B., Steinhilber D., Murganic B., Mijatović S., Maksimović-Ivanić D., Hey-Hawkins E. (2017). CarbORev-5901: The first carborane-based inhibitor of the 5-lipoxygenase pathway. ChemMedChem.

[9] Schwarze B., Jelača S., Welcke L., Maksimović-Ivanić D., Mijatović S., Hey-Hawkins E. (2019). 2,2’-Bipyridine-modified tamoxifen: A versatile vector for molybdacarboranes. ChemMedChem.

[10] Scholz M., Steinhagen M., Heiker J.T., Beck-Sickinger A.G., Hey-Hawkins E. (2011). Asborin inhibits Aldo/Keto reductase 1A1. ChemMedChem.

[11] Neumann W., Xu S., Sárosi M.-B., Scholz M.S., Crews B.C., Ghebreselasie K., Banerjee S., Marnett L.J., Hey-Hawkins E. (2016). *nido*-Dicarbaborate induces potent and selective inhibition of cyclooxygenase-2. ChemMedChem.

[12] Gozzi M., Schwarze B., Hey-Hawkins E. (2021). Preparing (metalla)carboranes for nanomedicine. ChemMedChem.

[13] Gozzi M., Murganic B., Drača D., Popp J., Coburger P., Maksimović-Ivanić D., Mijatović S., Hey-Hawkins E. (2019). Quinoline-conjugated ruthenacarboranes: Toward hybrid drugs with a dual mode of action. ChemMedChem.

[14] Simpson J.E., Gammoh N. (2020). The impact of autophagy during the development and survival of glioblastoma. Open Biol..

[15] Gallagher L.E., Radhi O.A., Abdullah M.O., McCluskey A.G., Boyd M., Chan E.Y.W. (2017). Lysosomotropism depends on glucose: A chloroquine resistance mechanism. Cell Death Dis..

[16] Varisli L., Cen O., Vlahopoulos S. (2020). Dissecting pharmacological effects of chloroquine in cancer treatment: Interference with inflammatory signaling pathways. Immunology.

[17] Motaln H., Koren A., Gruden K., Ramšak Ž., Schichor C., Lah T.T. (2015). Heterogeneous glioblastoma cell cross-talk promotes phenotype alterations and enhanced drug resistance. Oncotarget.

[18] Diao W., Tong X., Yang C., Zhang F., Bao C., Chen H., Liu L., Li M., Ye F., Fan Q. (2019). Behaviors of glioblastoma cells in in vitro microenvironments. Sci. Rep..

[19] Couto M., Mastandrea I., Cabrera M., Cabral P., Teixidor F., Cerecetto H., Viñas C. (2017). Small-molecule kinase-inhibitors-loaded boron cluster as hybrid agents for glioma-cell-targeting therapy. Chem. Eur. J..

[20] Couto M., García M.F., Alamón C., Cabrera M., Cabral P., Merlino A., Teixidor F., Cerecetto H., Viñas C. (2018). Discovery of potent EGFR inhibitors through the incorporation of a 3D-aromatic-boron-rich-cluster into the 4-anilinoquinazoline scaffold: Potential drugs for glioma treatment. Chem. Eur. J..

[21] Couto M., Alamón C., Nievas S., Perona M., Dagrosa M.A., Teixidor F., Cabral P., Viñas C., Cerecetto H. (2020). Bimodal therapeutic agents against glioblastoma, one of the most lethal forms of cancer. Chem. Eur. J..

[22] Wilkinson S.M., Gunosewoyo H., Barron M.L., Boucher A., McDonnell M., Turner P., Morrison D.E., Bennett M.R., McGregor I.S., Rendina L.M. (2014). The first CNS-active carborane: A novel P2 × 7 receptor antagonist with antidepressant activity. ACS Chem. Neurosci..

[23] Weyerhäuser P., Kantelhardt S.R., Kim E.L. (2018). Re-purposing chloroquine for glioblastoma: Potential merits and confounding variables. Front. Oncol..

[24] Giakoumettis D., Kritis A., Foroglou N. (2018). C6 cell line: The gold standard in glioma research. Hippokratia.

[25] Song Y., Xu H., Chen W., Zhan P., Liu X. (2015). 8-Hydroxyquinoline: A privileged structure with a broad-ranging pharmacological potential. MedChemComm.

[26] Madonna S., Béclin C., Laras Y., Moret V., Marcowycz A., Lamoral-Theys D., Dubois J., Barthelemy-Requin M., Lenglet G., Depauw S. (2010). Structure-activity relationships and mechanism of action of antitumor bis 8-hydroxyquinoline substituted benzylamines. Eur. J. Med. Chem..

[27] Yang Y., Zhou Z., Wei Z.-Z., Qin Q.-P., Yang L., Liang H. (2021). High anticancer activity and apoptosis- and autophagy-inducing properties of novel lanthanide (III) complexes bearing 8-hydroxyquinoline-N-oxide and 1,10-phenanthroline. Dalton Trans..

[28] Al-Bari M.A.A. (2015). Chloroquine analogues in drug discovery: New directions of uses, mechanisms of actions and toxic manifestations from malaria to multifarious diseases. J. Antimicrob. Chemother..

[29] Yoon Y.H., Cho K.S., Hwang J.J., Lee S.-J., Choi J.A., Koh J.-Y. (2010). Induction of lysosomal dilatation, arrested autophagy, and cell death by chloroquine in cultured ARPE-19 cells. Investig. Ophthalmol. Vis. Sci..

[30] Mauthe M., Orhon I., Rocchi C., Zhou X., Luhr M., Hijlkema K.-J., Coppes R.P., Engedal N., Mari M., Reggiori F. (2018). Chloroquine inhibits autophagic flux by decreasing autophagosome-lysosome fusion. Autophagy.

[31] Dikic I., Elazar Z. (2018). Mechanism and medical implications of mammalian autophagy. Nat. Rev. Mol. Cell Biol..

[32] Lau A.N., Vander Heiden M.G. (2020). Metabolism in the tumor microenvironment. Annu. Rev. Cancer Biol..

